# Characterization and visualization of murine coagulation factor VIII-producing cells in vivo

**DOI:** 10.1038/s41598-021-94307-0

**Published:** 2021-07-21

**Authors:** Morisada Hayakawa, Asuka Sakata, Hiroko Hayakawa, Hikari Matsumoto, Takafumi Hiramoto, Yuji Kashiwakura, Nemekhbayar Baatartsogt, Noriyoshi Fukushima, Yoichi Sakata, Katsue Suzuki-Inoue, Tsukasa Ohmori

**Affiliations:** 1 Department of Biochemistry, School of Medicine, Jichi Medical University, 3311-1 Yakushiji, Shimotsuke, Tochigi 329-0498 Japan; 2 Center for Gene Therapy Research, Jichi Medical University, 3311-1 Yakushiji, Shimotsuke, Tochigi 329-0498 Japan; 3 Center for Molecular Medicine, Jichi Medical University, 3311-1 Yakushiji, Shimotsuke, Tochigi 329-0498 Japan; 4 Department of Pathology, School of Medicine, Jichi Medical University, 3311-1 Yakushiji, Shimotsuke, Tochigi 329-0498 Japan; 5 Department of Clinical and Laboratory Medicine, Faculty of Medicine, University of Yamanashi, 1110 Shimokato, Chuo, Yamanashi 409-3898 Japan

**Keywords:** Biochemistry, Proteins, Blood proteins, Biological techniques, Gene expression analysis, Microarray analysis

## Abstract

Coagulation factors are produced from hepatocytes, whereas production of coagulation factor VIII (FVIII) from primary tissues and cell species is still controversial. Here, we tried to characterize primary FVIII-producing organ and cell species using genetically engineered mice, in which enhanced green fluorescent protein (EGFP) was expressed instead of the *F8* gene. EGFP-positive FVIII-producing cells existed only in thin sinusoidal layer of the liver and characterized as CD31^high^, CD146^high^, and lymphatic vascular endothelial hyaluronan receptor 1 (Lyve1)^+^. EGFP-positive cells can be clearly distinguished from lymphatic endothelial cells in the expression profile of the podoplanin^−^ and C-type lectin-like receptor-2 (CLEC-2)^+^. In embryogenesis, EGFP-positive cells began to emerge at E14.5 and subsequently increased according to liver maturation. Furthermore, plasma FVIII could be abolished by crossing *F8* conditional deficient mice with Lyve1-Cre mice. In conclusion, in mice, FVIII is only produced from endothelial cells exhibiting CD31^high^, CD146^high^, Lyve1^+^, CLEC-2^+^, and podoplanin^−^ in liver sinusoidal endothelial cells.

## Introduction

Blood coagulation factor VIII (FVIII), a procofactor in the process of blood coagulation, is catalyzed into an activated form (FVIIIa) at the site of vascular injury. FVIIIa binds to coagulation factor IX on the activated platelet surface and facilitates factor X activation in the intrinsic coagulation pathway^1^. Hemophilia A is a common congenital hemorrhagic disorder that is caused by FVIII deficiency and mainly affects men^2^. Because liver transplantation could increase FVIII levels in patients with hemophilia A, the liver is considered the source of plasma FVIII^3,4^. However, plasma FVIII levels are not affected by liver dysfunction^5^, suggesting an exceptional mode of protein production from the liver. It was reported that FVIII could be produced from hepatocytes and endothelial cells, but not Kupffer cells^6^. FVIII production is elevated in patients with cirrhosis, which is associated with increased overgrowth of FVIII-producing endothelial cells^5^. Moreover, studies on organ transplantation reported several other origins of circulating FVIII. Plasma FVIII levels were reportedly increased by transplantation of the lymph nodes, spleen cells, bone marrow, and lung in several animal models and in patients with hemophilia A^3,4,7–11^, suggesting the existence of FVIII production from extrahepatic organs.

Previous elegant studies using genetically engineered mice reported that the source of FVIII is restricted to endothelial cells because the conditional deletion of mouse genes encoding FVIII and mannose-specific lectin (*F8* and *Lman1*, respectively) in endothelial cells specifically reduced plasma FVIII levels^12,13^. *F8* messenger RNA (mRNA) expression has been detected in several organs^14^, and FVIII synthesis was observed from endothelial cells isolated from anatomically diverse organs such as liver sinusoidal endothelial cells (LSECs), glomerular endothelial cells, blood outgrowth endothelial cells, and lung microvascular endothelial cells^15–18^. FVIII and von Willebrand factor (VWF) proteins were also detected in Weibel–Palade bodies within glomerular microvascular endothelial cells and umbilical vein endothelial cells^19^. Furthermore, lymphatic endothelial cells were identified as a major cellular source of FVIII in extrahepatic tissues^20^. These data suggest that various types of endothelial cells from diverse organs are capable of expressing FVIII. However, the actual organs and the cellular characteristics of endothelial cells producing FVIII protein in vivo remain unclear. This study aimed to identify the organs and cells responsible for FVIII production using genetically engineered mice.

## Results

### Generation of FVIII knock-in mice

**Figure 1:**
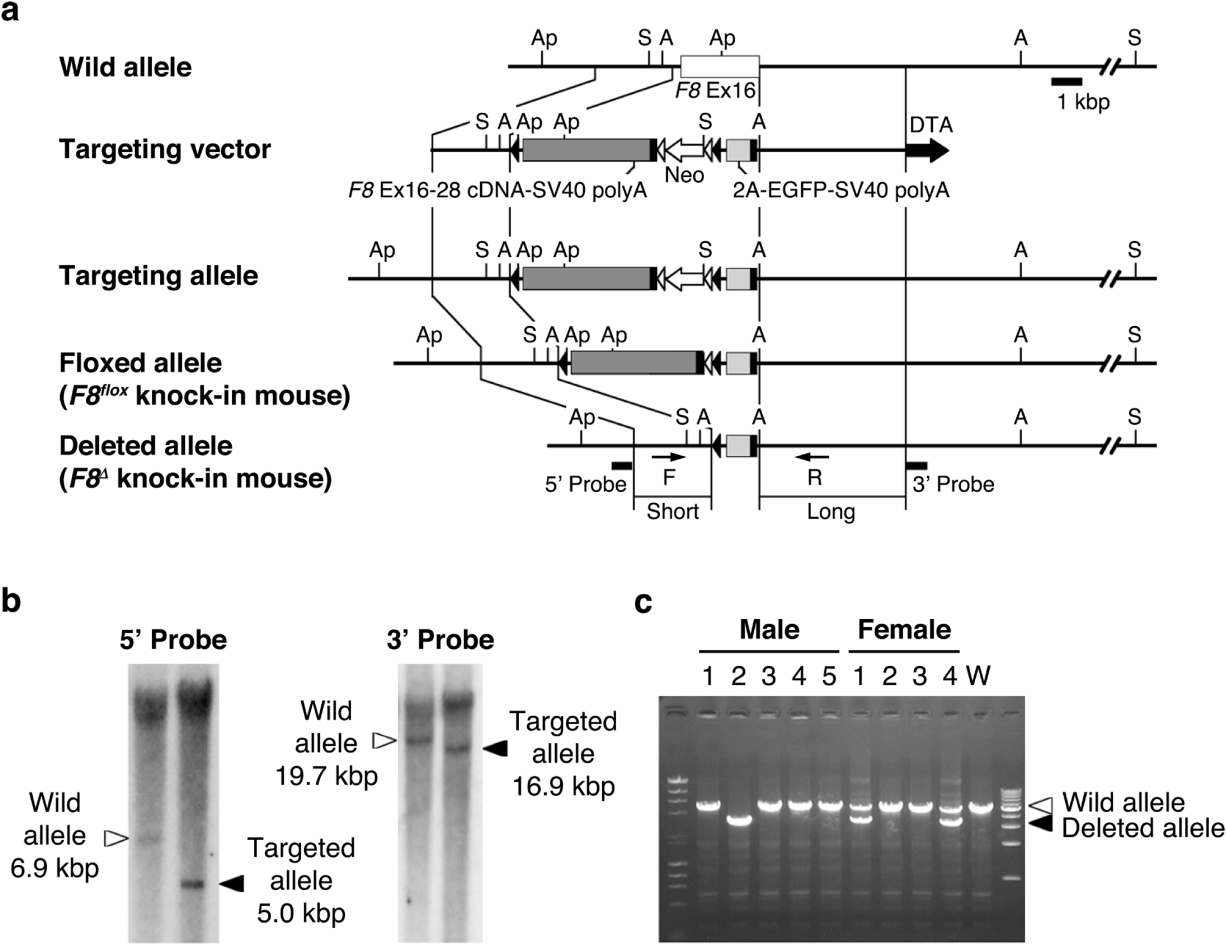
*F8* targeting strategy in *F8* knock-in mice. (**a**) Schematic of *F8* targeting construct. Diagrams show wild-type allele, targeting vector, targeting allele, floxed allele (*F8*^*flox*^ knock-in), and deleted allele (*F8*^*Δ*^ knock-in). The targeting vector was constructed by flanking the *F8* exons 16–28 cDNA-SV40 polyA and PGK-Neo cassette with *loxP* sites, followed by linking 2A-EGFP cDNA-SV40 polyA. White boxes, *F8* exon 16; dark gray boxes, *F8* cDNA including exons 16–28 (stop codon); light gray boxes, 2A-EGFP cDNA; black boxes, SV40 poly A; white arrow, PGK-Neo cassette; black triangles, loxP sites; white triangles, FRT sites; black arrow, DTA. Restriction enzyme sites: Ap, *Apa*LI; S, *Sph*I; A, *Apa*I. (**b**) Southern blotting of targeted embryonic stem cells. Genomic DNA was digested with *Apa*L I or *Sph* I, and probed with the 5′ or 3′ probe. Locations of probes are indicated in (**a**). (**c**) Confirmation of deletion of *F8* cDNA (exons 16–28) in *F8*^*Δ*^ knock-in mice. PCR was performed using the indicated F and R primers in (**a**).

We first generated *F8*^*flox*^ knock-in mice in which Cre induction led to the elimination of *F8* with *EGFP* expression (Fig. 1a). We further generated *F8*^*Δ*^ knock-in (*F8*^*Δ*^-KI) mice with the systemic deletion of *F8* by mating *F8*^*flox*^ knock-in mice with CAG-Cre mice (Fig. 1a–c) to identify the cells and organs responsible for the production of FVIII protein in vivo. The plasma FVIII:C level in *F8*^*Δ*^-KI mice was confirmed to be < 1%. Because the expression of *EGFP* in *F8*^*Δ*^-KI mice is regulated by the *F8* gene promoter, we used them to identify FVIII-producing cells by EGFP tracking in further experiments.

### Identification of FVIII-producing cells

**Figure 2:**
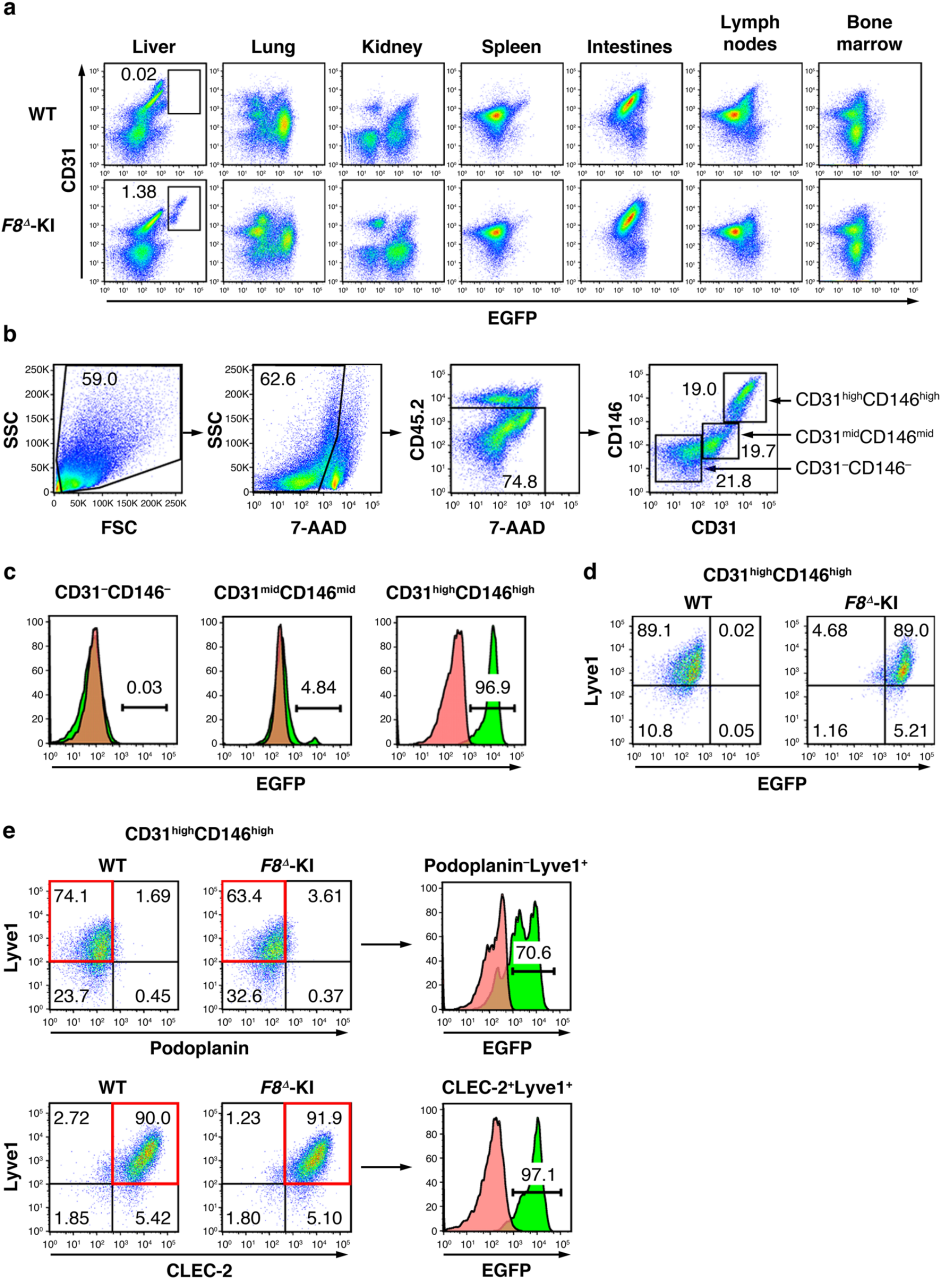
The characterization of EGFP-positive cells in *F8*^*Δ*^ knock-in mice. (**a**) Cells were isolated from the indicated organs in adult wild-type C57BL/6J male mice (WT) and *F8*^*Δ*^ knock-in (*F8*^*Δ*^-KI) mice. EGFP and CD31 expressions in the cells were determined by flow cytometry. Plots represent the expression levels of EGFP (horizontal) and CD31 (vertical) after the exclusion of dead cells. Boxes in the liver plots indicate the EGFP-positive population in WT and *F8*^*Δ*^-KI mice. (**b**) After the exclusion of dead cells and CD45^+^ cells, liver cells were fractioned into three populations according to the expression levels of CD31 (horizontal) and CD146 (vertical): CD31^–^CD146^–^, CD31^mid^CD146^mid^, and CD31^high^CD146^high^. (**c**) Based on the three populations in (**b**), EGFP expression in WT and *F8*^*Δ*^-KI mice was analyzed by histograms. Pink-filled and green-filled histograms indicate WT and *F8*^*Δ*^-KI mice, respectively. (**d**) Plots represent the expression levels of EGFP (horizontal) and Lyve1 (vertical) in CD31^high^CD146^high^ liver cells obtained from WT and *F8*^*Δ*^-KI mice. (**e**) Based on the CD31^high^CD146^high^ population in (**b**), podoplanin and CLEC-2 expressions were analyzed in WT and *F8*^*Δ*^-KI mice. The upper plots represent the expression levels of podoplanin (horizontal) and Lyve1 (vertical). The lower plots represent the expression levels of CLEC-2 (horizontal) and Lyve1 (vertical). Upper and lower histograms represent EGFP expression in podoplanin^–^Lyve1^+^ and CLEC-2^+^Lyve1^+^ populations, respectively. (**a**–**e**) The numbers in gated areas indicate the percentage of cells (n = 3).

We attempted to identify EGFP-positive organs responsible for FVIII production in *F8*^*Δ*^-KI mice by flow cytometry. We focused on CD31-positive endothelial cells in organs because endothelial cells are reported to be a major source of FVIII production^12,13^. CD31-expressing liver endothelial cells were the only cell population to express EGFP, and no EGFP-positive endothelial cells were detected in the lung, kidney, spleen, intestines, lymph nodes, or bone marrow (Fig. 2a). We further examined the characteristics of EGFP-positive endothelial cells in the liver. Liver endothelial cells were separated into three populations according to their CD31 and CD146 expression profile (Fig. 2b). EGFP expression in *F8*^*Δ*^-KI mice was mainly observed in the CD31^high^CD146^high^ population, but expression levels of 0.03% and 4.84% were observed in the CD31^–^CD146^–^ population and CD31^mid^CD146^mid^ population, respectively (Fig. 2c).

Lymphatic endothelial cells have previously been reported to be FVIII-producing cells^20^, and many of their cellular characteristics are common to sinusoidal endothelial cells. The EGFP-positive cells in CD31^high^CD146^high^ population also expressed Lyve1, a marker for sinusoidal endothelial cells and lymphatic endothelial cells (Fig. 2d). Low levels of Lyve1 expression were also observed in the CD31^mid^CD146^mid^ population with low EGFP expression (Supplementary Fig. S1a). We focused on the expression of podoplanin as a marker for lymphatic endothelial cells to identify the different characteristics between FVIII-producing cells and lymphatic endothelial cells^21^. Although mAb was useful to detect podoplanin in lung cells (Supplementary Fig. S1b), podoplanin was not expressed in the CD31^high^CD146^high^Lyve1^+^ population of the liver, and EGFP-positive cells were detected in the podoplanin^−^Lyve1^+^ population (Fig. 2e). LSECs reportedly express several members of CLEC members^22–24^. EGFP-positive cells in the CD31^high^CD146^high^Lyve1^+^ population expressed CLEC-2, a physiological receptor for podoplanin, abundantly expressed on platelets^25^ (Fig. 2e). These data suggest that FVIII-producing sinusoidal endothelial cell in the liver can be clearly distinguished from lymphatic endothelial cells in the expression profile of podoplanin and CLEC-2.

### Localization of FVIII-producing cells

**Figure 3:**
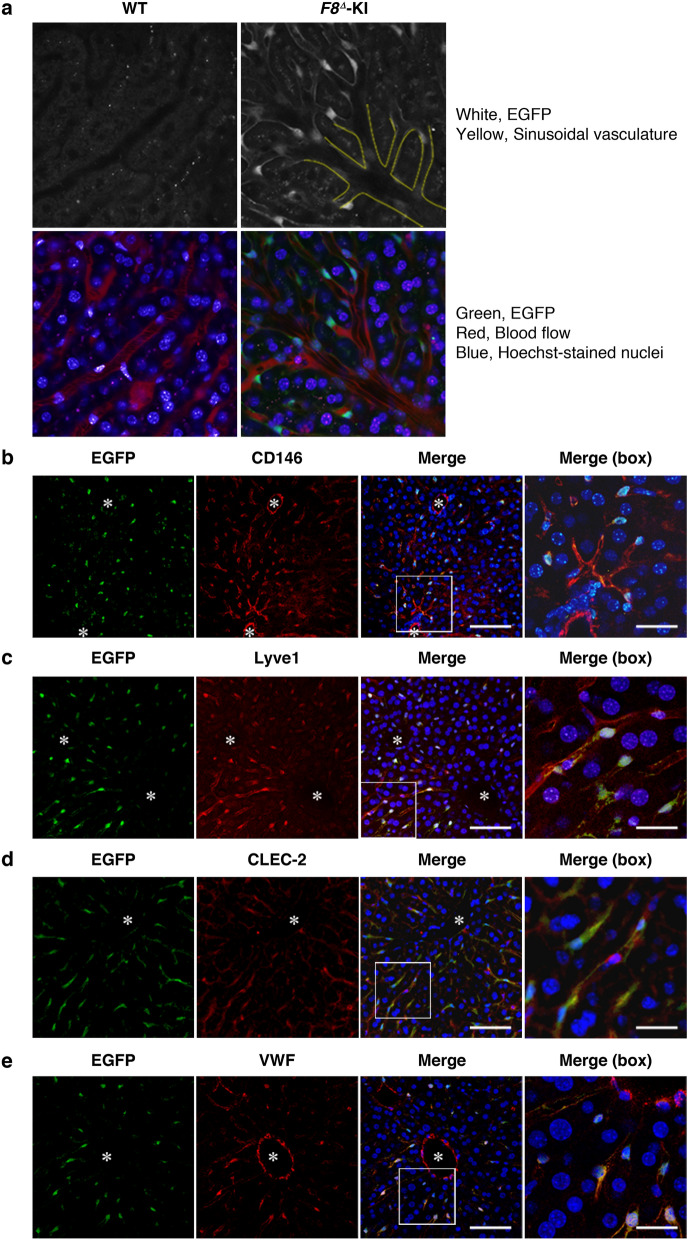
Histological localization of EGFP-positive cells in *F8*^*Δ*^ knock-in mice. (**a**) Intravital imaging in the liver of adult WT and *F8*^*Δ*^-KI male mice was observed by intravital confocal microscopy. Red, blood flow; blue, Hoechst-stained nuclei; white (upper) and green (lower), EGFP. Yellow lines in the top panel indicate thick sinusoidal vasculature. (**b**–**e**) Liver sections obtained from adult *F8*^*Δ*^-KI mice were subjected to immunofluorescence staining for EGFP and CD146 (**b**), EGFP and Lyve1 (**c**), EGFP and CLEC-2. (**d**), and EGFP and VWF. (**e**) Nuclear localization was simultaneously examined by DAPI staining. Confocal microscopy was used to observe the localization of each antigen, nucleus, and their merged image. The magnifications of the boxed areas in the merged image are shown in the right panel. Scale bars: 75 μm in merged images and 25 μm in magnification of the merged images; *central vein (n = 3).

We employed immunohistochemistry for EGFP expression to identify the histological localization of FVIII-producing cells. Immunohistochemical analysis did not detect EGFP expression in liver parenchymal cells (hepatocytes) or around the central vein (Supplementary Fig. S2). Intravital microscopy clearly identified EGFP expression in the liver of *F8*^*Δ*^-KI mice, revealing that EGFP-positive cells were localized to liver endothelial cells constructing thin sinusoids (Fig. 3a, Supplementary Movies 1 and 2).

We stained liver sections with antibodies against endothelial markers, such as CD146, Lyve1, CLEC-2, and VWF (Fig. 3b–e and Supplementary Fig. S3), to further determine the profile of EGFP-positive cells in the liver. CD146-positive endothelial cells were found to be present not only in the sinusoid but also in the central veins (Fig. 3b). Lyve1 and CLEC-2 were only expressed in sinusoidal endothelial cells, whereas the dominant expression of VWF was observed in the central veins (Fig. 3c–e). Taken together, it was observed that EGFP-positive cells localized in the sinusoids and coexpressed CD146, Lyve1, CLEC-2, and VWF.

### FVIII-producing cells during development

**Figure 4:**
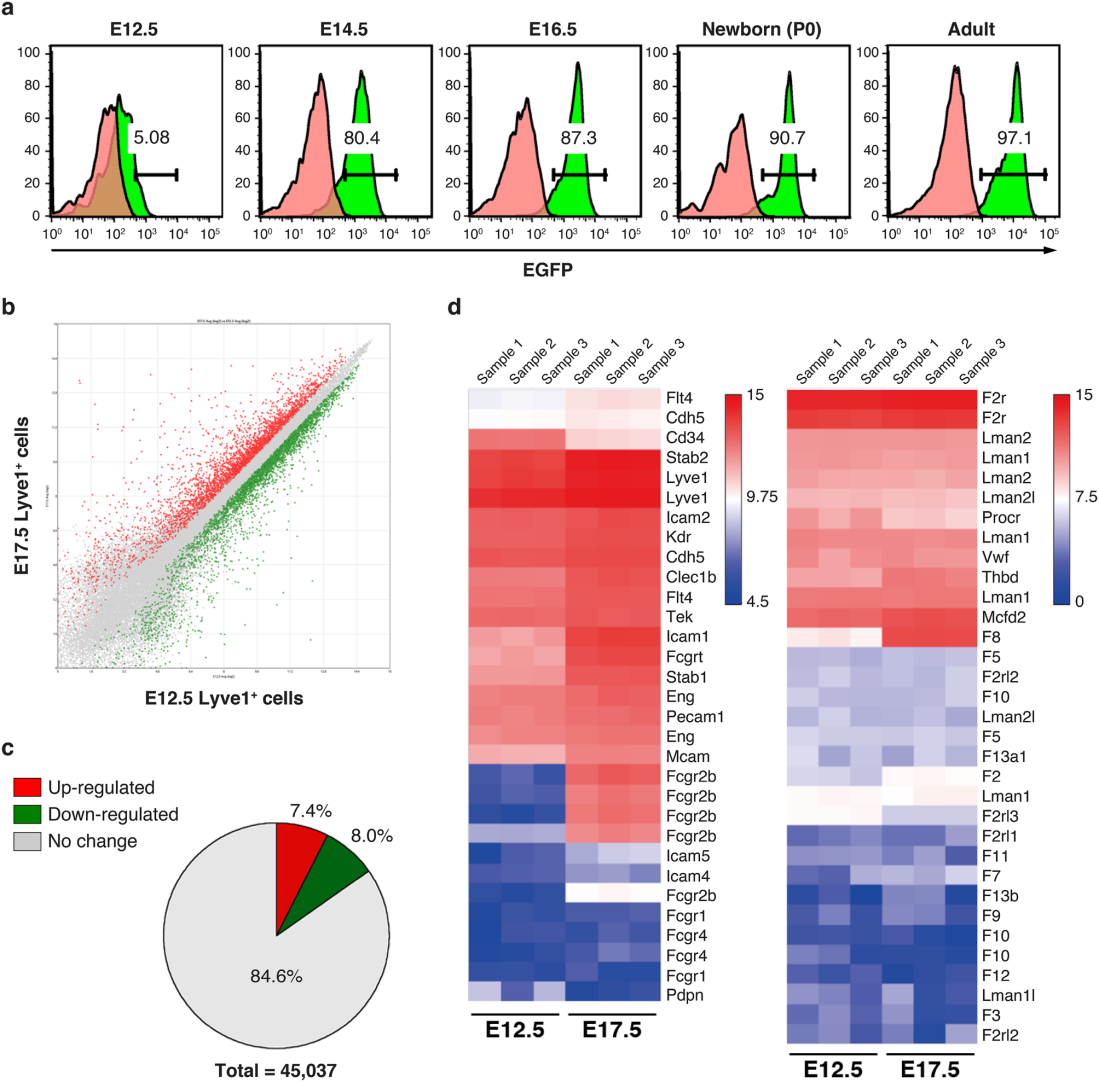
Microarray analysis of Lyve1-positive cells in liver during embryonic development. (**a**) Liver cells were obtained from fetal (E12.5, E14.5, and E16.5), newborn (P0), and adult WT and *F8*^*Δ*^-KI mice. After the exclusion of dead cells and CD45^+^ cells, EGFP expression was detected in CD31^high^CD146^high^Lyve1^+^CLEC-2^+^ population. Pink-filled and green-filled histograms indicate WT and *F8*^*Δ*^-KI mice, respectively. The numbers in gated areas indicate the percentage of cells (n = 3). (**b**) Lyve1-positive cells were isolated as CD31^high^CD146^high^Lyve1^+^ cells from the liver of C57BL/6J fetal mice (E12.5 and E17.5) (n = 3). Scatter plot of differentially expressed genes in E12.5 Lyve1^+^ cells and E17.5 Lyve1^+^ cells. Red and green dots indicate upregulated and downregulated genes with a greater than twofold change, respectively. (**c**) Percentage of upregulated and downregulated genes with a greater than twofold change. (**d**) Heatmap of differentially expressed genes related to coagulation factors and endothelial cell markers between E12.5 Lyve1^+^ cells and E17.5 Lyve1^+^ cells. Blue and red colors represent low and high expression levels, respectively.

We next investigated EGFP expression in the liver during the embryonic development of *F8*^*Δ*^-KI mice (Fig. 4a). No EGFP expression was detected in the whole fetus at embryonic day (E)10.5. Of note, EGFP expression in CD31^high^CD146^high^Lyve1^+^CLEC-2^+^ liver endothelial cells surged from 5% at E12.5 to 80% at E14.5. Furthermore, its expression reached more than 90% at birth (Fig. 4a). These results suggest that FVIII in CD31^high^CD146^high^Lyve1^+^CLEC-2^+^ liver endothelial cells expressed in the later phase of embryonic development.

We further examined mRNA expression profiles in Lyve1-positive cells during embryonic development. We isolated CD31^high^CD146^high^Lyve1^+^ cells from the liver obtained from E12.5 and E17.5 mouse embryos and compared mRNA expressions by microarray analysis (Supplementary Fig. S4a and Fig. 4b). Of 45,037 probes examined, 8.0% and 7.4% of the genes were found to be significantly decreased or increased more than twofold, respectively (Fig. 4c). We analyzed the expression of coagulation factors and endothelial markers. We confirmed the decrease in CD34 expression and significant increase in *Fcgr2b* and *F8* (Fig. 4d). The genes that were upregulated at E17.5 also included *Mcam* (CD146) and *Lyve1*, which were used as cell surface markers for cell sorting. The expressions of other coagulation factors and *Vwf* were not changed. We also analyzed *Clec* family members and found dominant expression of *Clec2d*, *Clec14a*, *Clec1b*, and *Clec4g* (Supplementary Fig. S4b). The upregulated genes such as *Clec1b*, *Clec4g*, and *Stab2* were also specifically expressed in CD31^high^CD146^high^Lyve1^+^ liver endothelial cells of adult mice (Supplementary Fig. S5).

### Conditional deletion of F8 in Lyve1-positive endothelial cells

**Figure 5:**
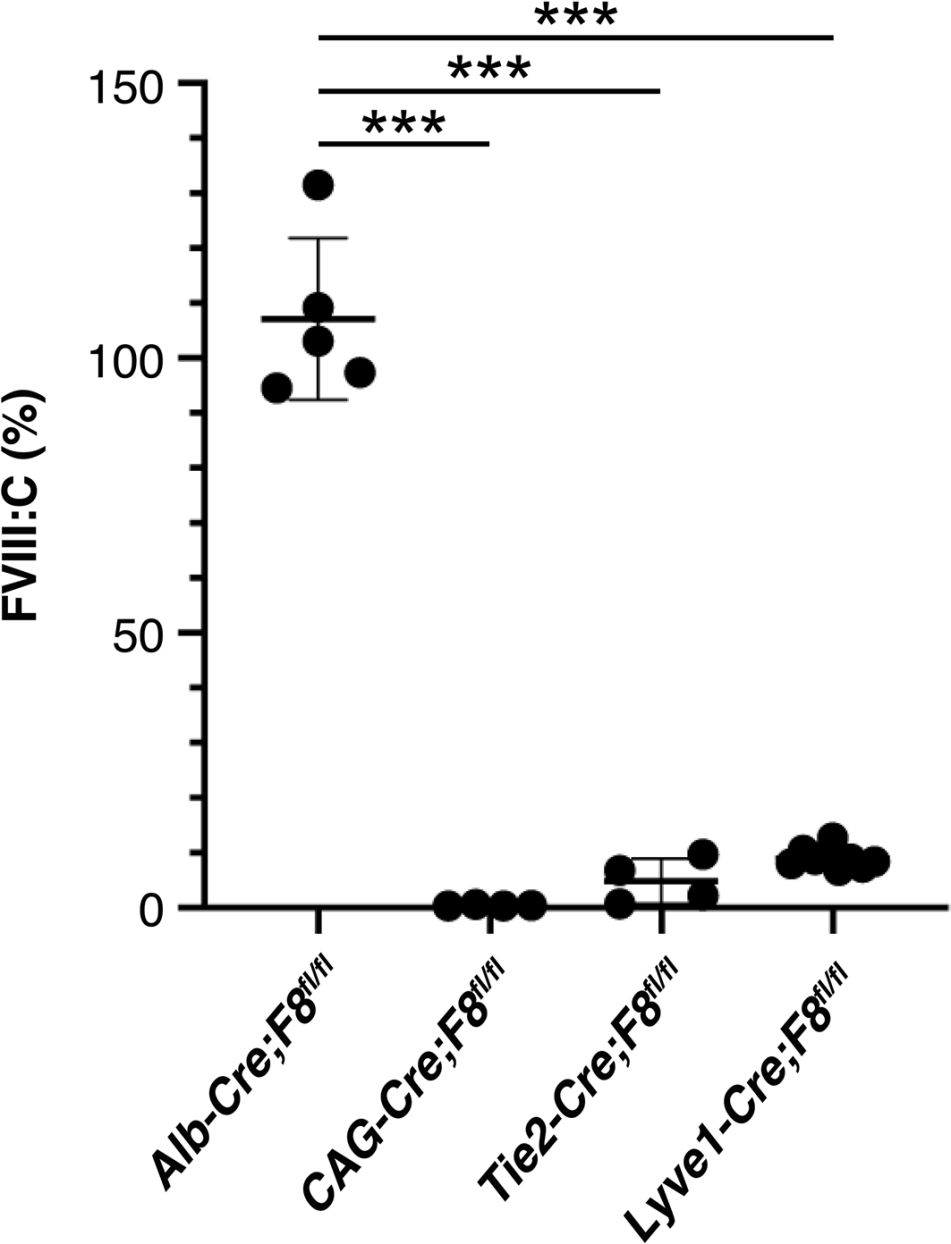
Decrease in plasma FVIII activity by the conditional deletion of *F8*. Plasma levels of FVIII activity (FVIII:C) were measured by one-stage clotting assay in the conditional knockout *F8* male mice obtained by crossing *F8 *^*fl/fl*^ mice with *Alb-Cre*, *CAG-Cre*, *Tie2-Cre*, or *Lyve1-Cre* mice. Dots represent plasma FVIII:C levels in each mouse. Bars are means ± SEM (n = 4–8). ***P < 0.0001 (2-tailed Student’s *t*-test).

Previous reports suggested mating with Tie2-Cre mice, but not Alb-Cre mice, suppressed FVIII:C expression in *F8* conditional deficient mice^12,13^. We crossed *F8* conditional deficient mice with Lyve1-Cre mice to further confirm the importance of sinusoidal endothelial cells for FVIII production. Because Lyve1 is expressed in sinusoidal endothelial cells but not vascular endothelial cells^26^, Lyve1 promoter enables to more specifically express Cre in sinusoidal endothelial cells than Tie2 promoter. We employed *F8*^*fl/fl*^ conditional deficient mice (*F8*^*tm1Rmnt/J*^) possessing 2 *loxP* sites flanking exons 17–18^13^. Male offspring of crosses between female *F8*^*fl/fl*^ conditional deficient mice and male Lyve1-Cre mice showed a marked decrease in FVIII:C by Cre expression compared with male littermates without Cre expression (Fig. 5). These data clearly support our idea that FVIII was produced predominantly from LSECs.

## Discussion

Details of the exact organ and endothelial cell types involved in FVIII synthesis have been controversial for decades^3,4,7–9,15–18^. To resolve this issue, we generated *F8*^*flox*^ knock-in mice in which Cre induction led to the elimination of *F8* and expression of *EGFP* to aid the identification of organs and cells responsible for FVIII production. After the systemic induction of Cre, we could clearly identify FVIII-producing cells as EGFP-positive cells in mouse.

We found that the liver was the only organ to produce FVIII*,* and sinusoidal endothelial cells exhibiting CD31^high^, CD146^high^, Lyve1^+^, and CLEC-2^+^ were responsible for FVIII production. This endothelial fraction was not observed in other organs, such as the lung, kidney, spleen, intestines, lymph nodes, and bone marrow. Although previous observation revealed that lymphatic endothelial cells were the major source of extrahepatic FVIII production^20^, EGFP-positive cells could not be detected in lymph nodes and spleen in this study. Our findings are consistent with the fact that the FVIII:C of patients with hemophilia A reaches a normal range soon after liver transplantation^3,4^. Sinusoidal endothelial cells and lymphatic endothelial cells substantially share similar cellular and phenotypic characteristics, for example, minimal basement membranes and expression profile of Lyve1^+^, VSP-1^+^, and Reelin^+^ and CD34^−^^27^. In addition, some members of C-type lectin receptor family such as CLEC-4G and CLEC-4M are expressed in both cells^23,24^. In contrast, the FVIII-producing sinusoidal endothelial cells could be completely distinguished from lymphatic endothelial cells by expression profiles of podoplanin and CLEC-2; sinusoidal endothelial cells express CLEC-2, whereas lymphatic endothelial cells express its ligand podoplanin. It is very interesting that two molecules acting as receptor-ligand relationship could separate the characteristics between these cells. Podoplanin on lymphatic endothelial cells plays an essential role for the blood/lymphatic separation and lung development through the interaction with CLEC-2 on platelet surface^25^. Further analysis will be required to identify the ligand for CLEC-2 on sinusoidal endothelial cells because the physiological role of CLEC-2 on sinusoidal endothelial cells still remains unknown.

We did not observe FVIII-producing endothelial cells until E12.5 and found that sinusoidal endothelial cells expressing FVIII surged at E14.5 during embryonic development. These data suggest that the FVIII production system matures in the later phase of liver development. EGFP expression profile in sinusoidal endothelial cells just after birth was similar to those in adult liver. This is consistent with previous clinical observation that plasma FVIII:C in newborn is the same as the adult, whereas levels of vitamin K-dependent coagulation factors at birth are significantly lower than those of adults^28^. Sinusoidal development follows a series of processes^29^. First, sinusoidal endothelial cells are lined by a laminin-rich basal membrane, then start to fenestrate by stereotypic differentiation at E14–15, and finally undergo maturation of their anatomical structure^29,30^. Our investigation of the profile of FVIII-producing cells could help identify the factor(s) responsible for sinusoid maturation because the expression of FVIII seems to occur during the later phase.

VWF and FVIII circulate within the blood as a tight complex in which VWF stabilizes the structure of FVIII and protects it from proteolytic degradation^31^. It was previously reported that VWF is mainly expressed in capillary endothelial cells but not in lymphatic endothelial cells, whereas FVIII is expressed in lymphatic endothelial cells, suggesting a differential pattern of expression between the proteins^20^. However, EGFP expression by the *F8* gene promoter was not observed in lymphatic endothelial cells in this study. We observed predominant VWF expression in endothelial cells of the central vein; hence, our data also suggest that vascular endothelial cells are the main source of circulating VWF. However, it should be noted that most VWF molecules exist in a nonbinding form with FVIII in the circulation because plasma concentration of VWF is significantly higher than those of FVIII (50 nM and 1 nM, respectively)^32^. In this study, sinusoidal endothelial cells also expressed VWF at lower levels, and VWF colocalized with EGFP-positive cells in *F8*^*Δ*^ knock-in mice. It is conceivable that formation of the FVIII/VWF complex occurs within the cells. Indeed, human endothelial cells stimulated with vasopressin in vitro secreted cell-anchored ultralarge VWF strings covered with bound FVIII^19^. Further analysis is needed to clarify the involvement of VWF in the endothelial cell secretion of FVIII.

The current adeno-associated virus (AAV) vector-mediated gene therapy for hemophilia expresses the target coagulation factor in hepatocytes^33–35^, which are non-physiological cells for producing FVIII. A recent study revealed that the plasma activity of FVIII ectopically expressed from hepatocytes by AAV-mediated gene therapy was 1.3- to 2.0-fold higher in a one-stage coagulation assay than in a chromogenic substrate assay^36^. The *F8* gene sequence is identical, indicating that FVIII has undergone unique post-transcriptional modifications in hepatocytes. Ectopic post-transcriptional modifications have been suggested to affect the folding, trafficking, and secretion of FVIII in hepatocytes and alter cell viability and function via endoplasmic reticulum stress^37^. Moreover, FVIII expression in hemophilia A gene therapy tends to gradually decrease over time compared to that in hemophilia B. Hemophilia A gene therapy also required the administration of huge amounts of AAV vectors in clinical trials, even though the molar concentration of FVIII is much lower than that of FIX^33^. Gene therapy targeting FVIII-producing cells may resolve these problems. Liver sinusoid endothelial cells are almost quiescent under the physiological state^38^, representing an ideal target for AAV-mediated gene therapy. Therefore, we are currently developing strategies to efficiently introduce AAV vectors into FVIII-producing cells in vivo for hemophilia A gene therapy and genome editing. Furthermore, it has been reported that the administration of sinusoidal endothelial cells expressing FVIII generated from patient-derived induced pluripotent stem cells (iPSCs) improved the hemophilia A phenotype in mice^39^. Deriving sinusoidal endothelial cells from the patient’s gene-corrected iPSCs may also provide an attractive source for cell-based hemophilia A therapy.

This study has some limitations. First, we must consider the species differences to extrapolate the implications of this study to human biology. It is possible that the mechanism of FVIII synthesis differs among species. Indeed, several reports suggest the existence of a compensatory mechanism by other organs to maintain the plasma FVIII. In a porcine model of fulminant hepatic failure caused by a total hepatectomy, plasma FVIII levels increased in the absence of the liver^40^. In addition, previous observations in dogs and humans suggest that the liver may not be the unique organ that produces FVIII. In fact, when the liver of a hemophilic dog was transplanted into a normal dog, the recipient did not develop the same phenotype as the hemophilic animal^41^. In humans, FVIII production was sustained in a transplant recipient of a hemophilia A donor liver^42^. Second, our study clearly identified FVIII-producing cells in vivo, but we could not assess the cellular localization and release mechanism of FVIII. Because we can only look at EGFP protein instead of FVIII, our data could not consider the protein interaction of FVIII. FVIII localization within the cells might be changed by the interaction with other proteins. Although previous reports suggest that FVIII is packaged with VWF in Weibel–Palade bodies in endothelial cells^19^, the existence of these bodies in sinusoidal endothelial cells is controversial^43^.

In conclusion, this study suggested that FVIII in mice is mainly produced from LSECs with expressions of CD31^high^, CD146^high^, Lyve1^+^, and CLEC-2^+^. Moreover, we found that FVIII production in the liver starts and matures during the later phase of embryonic development. These data help resolve the controversy about which organ-derived endothelial cell types synthesize FVIII. The next challenge will be to elucidate the precise mechanism by which FVIII release is regulated, with the ultimate aim of developing gene therapy to efficiently transduce FVIII-producing cells in vivo.

## Methods

### Animals

Wild-type C57BL/6J (WT) mice were purchased from Japan SLC (Shizuoka, Japan). *F8*^*fl/fl*^ mice possessing 2 *loxP* sites flanking exons 17–18 (*F8*^*tm1Rmnt/J*^)^13^, Alb-Cre mice (B6.Cg-Tg(Alb-Cre)21Mgn/J)^44^, and lymphatic vascular endothelial hyaluronan receptor-1 (Lyve1)-Cre (B6;129P2-*Lyve1*^*tm1.1(EGFP/cre)Cys*^/J) mice^45^ were obtained from The Jackson Laboratory (Sacramento, CA, USA). Tie-2-Cre mice (B6.Cg-Tg(Tek-Cre)1Ywa)^46^ and CAG-Cre mice (B6.Cg-Tg(CAG-Cre)CZ-MO2Osb)^47^ were obtained from RIKEN BRC (Ibaraki, Japan). The Institutional Animal Care and Concern Committee of Jichi Medical University approved all animal procedures (Approval Number: 17117-13), and animal care was performed according to the committee’s guidelines and as per ARRIVE Guidelines/Checklist.

### Generation of F8 knock-in mice

We generated a targeting vector containing a floxed *F8* cDNA sequence (exons 16–28), SV40 polyA sequence, and PGK-Neo cassette, followed by linking a 2A-EGFP cDNA and SV40 polyA sequence. Sequences of the 5′ arm (3.0 kb) and 3′ arm (5.6 kb) of *F8* were amplified by polymerase chain reaction (PCR) and then inserted into the above construct. The linearized targeting vector was then transduced into mouse embryonic stem cells (ESGRO Complete Adapted C57/BL6 mouse embryonic stem cell Line, Merck, Darmstadt, Germany) by electroporation. To generate chimeric mice, the embryonic stem cells were injected into mouse blastocysts after the confirmation of gene targeting by Southern blotting. Chimeric mice were bred with C57BL/6 mice to produce F1 mice. The F1 mice were bred with CAG-FLPe transgenic mice (*F8*^*flox*^ knock-in mice) to delete the PGK-Neo cassette. To generate a systemic deletion of *F8* (flox to Δ), *F8*^*flox*^ knock-in mice were crossed with CAG-Cre transgenic mice to produce *F8*^*Δ*^ knock-in mice*.* Deletion of *F8* cDNA (exons 16–28) was confirmed by PCR analysis using the following primers: F 5′-GTATGTATATGTGTCAGCAAATATGC-3′, R 5′-ATAATTTCACACCATAAGGCCAATA-3′.

### Dissociation of cells from primary tissue and flow cytometry

Mice anesthetized with isoflurane were perfused with a perfusion buffer (10 mM HEPES, 140 mM NaCl, 6.5 mM KCl, 5 mM CaCl_2_, pH 7.4) and their tissues were removed. Bone marrow cells and spleen cells were isolated as previously described^48^. Other solid tissues were minced into 3–4 mm pieces with scissors and then incubated with 20 µg/mL of Collagenase/Dispase (Roche Applied Science, Mannheim, Germany) and 10 µg/mL of DNase I (Sigma-Aldrich, Louis, MO, USA) for 30 min. Endothelial cells were separated by 25/50% Percoll gradient centrifugation and washed with phosphate-buffered saline (PBS) containing fetal bovine serum.

The expression of EGFP and specific antigens was detected using flow cytometry. Isolated cells were preincubated with an anti-CD16/CD32 mAb to block the Fc receptor (TruStain FcX; BioLegend, Inc., San Diego, CA, USA) and then incubated with fluorescent-conjugated antibodies for 30 min at 4 °C. The following antibodies were used for analyses: anti-CD45.2 mAb conjugated with violetFluor 450 (clone 104; Tonbo Biosciences, San Diego, CA, USA), anti-CD31 mAb conjugated with PE/Cy7 (clone 390), anti-CD146 mAb conjugated with PerCP/Cy5.5 or PE (clone ME-9F1), anti-podoplanin mAb conjugated with PE (clone 8.1.1), anti-CLEC-2 (clone 17D9) conjugated with PE (all BioLegend), and anti-Lyve1 mAb conjugated with eFluor 660 (clone ALY7; Thermo Fisher Scientific, Waltham, MA, USA). Just before analysis, 7-amino-actinomycin D (7-AAD) (BioLegend) or fixable viability dye eFluor 506 (FVD506) (Thermo Fisher Scientific) was added to the samples to detect dead cells. A BD LSRFortessa flow cytometer (BD Biosciences, Franklin Lakes, NJ, USA) was used to analyze cellular fluorescence, and FlowJo software (BD Biosciences) was used for data analysis.

### Immunohistochemical analysis

Mice anesthetized with isoflurane were perfused with 50 mL PBS. For double staining of EGFP with endothelial markers, liver tissues were fixed with 4% paraformaldehyde, incubated with PBS containing sucrose (10–20%), and then frozen in the presence of Tissue-Tek O.C.T. Compound (Sakura Fintek Japan, Tokyo, Japan) in dry ice/ethanol. Tissue sections were blocked with 5% donkey serum and then incubated with an anti-EGFP polyclonal antibody (MBL Co.) and a specific antibody for endothelial markers as follows: anti-CD31 mAb (clone 390; BioLegend), anti-CD146 mAb (clone ME-9F1; BioLegend), anti-CLEC-2 mAb (clone 2A2B10; kindly provided from Dr. Suzuki-Inoue), anti-Lyve1 mAb (clone ALY7; Thermo Fisher Scientific), or anti-VWF polyclonal antibody (code ab11713; Abcam, Cambridge, UK). Sections were then incubated with a species-specific secondary antibody conjugated with Alexa Fluor 594 (Thermo Fisher Scientific) and anti-rabbit IgG conjugated with Alexa Fluor 488 (for the detection of EGFP) for 2 h at 4 °C. Slides were mounted with VECTASHIELD Mounting Medium with DAPI (Vector Laboratories, Burlingame, CA, USA). Immunofluorescence staining was observed and photographed using a confocal microscope (Leica TCS SP8; Leica Microsystems, Wetzlar, Germany).

### Intravital microscopy

Intravital microscopy was performed as reported previously^49^. Briefly, anesthetized mice were injected with rhodamine B isothiocyanate-Dextran (5 mg/body; Sigma-Aldrich) and Hoechst 33342 (3 mg/body; Thermo Fisher Scientific). A resonance scanning confocal microscope (Nikon A1R; Nikon, Tokyo, Japan) was used to obtain sequential images of the liver.

### Isolation of liver endothelial cells of fetal mice

C57BL/6J fetus at E12.5 and E17.5 were sacrificed, and then livers were removed. After treatment of liver tissues with collagenase/dispase and DNase I, the cells were incubated with anti-CD16/CD32 antibody and then stained with fluorescent-labeled antibodies against CD31, CD146, and Lyve1. Dead cells were stained with 7-AAD. A BD FACSAriaII Special Order Research Product (BD Biosciences) was used to isolate the CD31^high^CD146^high^Lyve1^+^ cells.

### Microarray analysis

The total RNAs were isolated from CD31^high^CD146^high^Lyve1^+^ cells in fatal liver (E12.5 and E17.5) by NucleoSpin RNA kit (MACHEREY-NAGEL GmBH & Co, Duren, Germany). mRNA expressions were analyzed by GeneChip Mouse Genome 430 2.0 Array (Thermo Fisher Scientific) and detected by GeneChip Scanner 3000 7G (Thermo Fisher Scientific). Transcriptome Analysis Console software (Thermo Fisher Scientific) was used for data analysis.

### Measurement of FVIII activity

FVIII activity (FVIII:C) was measured using an activated partial thromboplastin time (APTT)-based one-stage clotting-time assay on an automated coagulation analyzer (Sysmex CA-500 or CS-1600 analyzer; Sysmex Corp., Kobe, Japan). Clotting time was measured by APTT (Thrombocheck APTT, Sysmex Corp.) after mixing the test mouse plasma with human FVIII-deficient plasma (Thrombocheck FVIII, Sysmex Corp.). FVIII:C was determined based on a calibration curve prepared using pooled plasma from wild-type C57BL/6J mice.

### Statistical analyses

Data are presented as means ± standard error of the mean of at least three independent experiments. Statistical significance was determined by 2-tailed Student’s *t* tests using GraphPad Prism version 8 (GraphPad Software, San Diego, CA, USA) or Microsoft Excel (Microsoft, Redmond, WA, USA). A value of *P* < 0.05 was considered to be statistically significant.

## Supplementary Information

Supplementary Information 1.

Supplementary Information 2.

## Data Availability

The original data in this study are available upon request from the corresponding author.

## References

[1] Thompson AR (2003). Structure and function of the factor VIII gene and protein. Semin. Thromb. Hemost..

[2] Hoyer LW (1994). Hemophilia A. N. Engl. J. Med..

[3] Bontempo FA (1987). Liver transplantation in hemophilia A. Blood.

[4] Marchioro TL, Hougie C, Ragde H, Epstein RB, Thomas ED (1969). Hemophilia: Role of organ homografts. Science.

[5] Hollestelle MJ, Geertzen HG, Straatsburg IH, van Gulik TM, van Mourik JA (2004). Factor VIII expression in liver disease. Thromb. Haemost..

[6] Do H, Healey JF, Waller EK, Lollar P (1999). Expression of factor VIII by murine liver sinusoidal endothelial cells. J. Biol. Chem..

[7] Groth CG (1974). Correction of coagulation in the hemophilic dog by transplantation of lymphatic tissue. Surgery.

[8] Veltkamp JJ (1974). Extrahepatic factor VIII synthesis. Lung transplants in hemophilic dogs. Transplantation.

[9] Liu L, Xia S, Seifert J (1994). Transplantation of spleen cells in patients with hemophilia A. A report of 20 cases. Transpl. Int..

[10] Aronovich A (2006). Correction of hemophilia as a proof of concept for treatment of monogenic diseases by fetal spleen transplantation. Proc. Natl Acad. Sci. U. S. A..

[11] Follenzi A, Raut S, Merlin S, Sarkar R, Gupta S (2012). Role of bone marrow transplantation for correcting hemophilia A in mice. Blood.

[12] Everett LA, Cleuren AC, Khoriaty RN, Ginsburg D (2014). Murine coagulation factor VIII is synthesized in endothelial cells. Blood.

[13] Fahs SA, Hille MT, Shi Q, Weiler H, Montgomery RR (2014). A conditional knockout mouse model reveals endothelial cells as the principal and possibly exclusive source of plasma factor VIII. Blood.

[14] Dashty M (2012). Characterization of coagulation factor synthesis in nine human primary cell types. Sci. Rep..

[15] Hollestelle MJ (2001). Tissue distribution of factor VIII gene expression in vivo—A closer look. Thromb. Haemost..

[16] Jacquemin M (2006). FVIII production by human lung microvascular endothelial cells. Blood.

[17] van den Biggelaar M (2009). Storage and regulated secretion of factor VIII in blood outgrowth endothelial cells. Haematologica.

[18] Shahani T (2014). Human liver sinusoidal endothelial cells but not hepatocytes contain factor VIII. J. Thromb. Haemost..

[19] Turner NA, Moake JL (2015). Factor VIII is synthesized in human endothelial cells, packaged in Weibel–Palade bodies and secreted bound to ULVWF strings. PLoS One.

[20] Pan J (2016). Patterns of expression of factor VIII and von Willebrand factor by endothelial cell subsets in vivo. Blood.

[21] Breiteneder-Geleff S (1999). Angiosarcomas express mixed endothelial phenotypes of blood and lymphatic capillaries: Podoplanin as a specific marker for lymphatic endothelium. Am. J. Pathol..

[22] Chaipan C (2006). DC-SIGN and CLEC-2 mediate human immunodeficiency virus type 1 capture by platelets. J. Virol..

[23] Liu W (2004). Characterization of a novel C-type lectin-like gene, LSECtin: Demonstration of carbohydrate binding and expression in sinusoidal endothelial cells of liver and lymph node. J. Biol. Chem..

[24] Pöhlmann S (2001). DC-SIGNR, a DC-SIGN homologue expressed in endothelial cells, binds to human and simian immunodeficiency viruses and activates infection in trans. Proc. Natl Acad. Sci. U. S. A..

[25] Suzuki-Inoue K (2018). Platelet CLEC-2: Roles beyond hemostasis. Semin. Thromb. Hemost..

[26] Si-Tayeb K, Lemaigre FP, Duncan SA (2010). Organogenesis and development of the liver. Dev. Cell.

[27] Nonaka H, Tanaka M, Suzuki K, Miyajima A (2007). Development of murine hepatic sinusoidal endothelial cells characterized by the expression of hyaluronan receptors. Dev. Dyn..

[28] Andrew M (1987). Development of the human coagulation system in the full-term infant. Blood.

[29] Ober EA, Lemaigre FP (2018). Development of the liver: Insights into organ and tissue morphogenesis. J. Hepatol..

[30] Enzan H (1997). Development of hepatic sinusoidal structure with special reference to the Ito cells. Microsc. Res. Tech..

[31] Lenting PJ, Van Schooten CJ, Denis CV (2007). Clearance mechanisms of von Willebrand factor and factor VIII. J. Thromb. Haemost..

[32] Terraube V, O’Donnell JS, Jenkins PV (2010). Factor VIII and von Willebrand factor interaction: Biological, clinical and therapeutic importance. Haemophilia.

[33] Rangarajan S (2017). AAV5-factor VIII gene transfer in severe hemophilia A. N. Engl. J. Med..

[34] George LA (2017). Hemophilia B gene therapy with a high-specific-activity factor IX variant. N. Engl. J. Med..

[35] Ohmori T, Mizukami H, Ozawa K, Sakata Y, Nishimura S (2015). New approaches to gene and cell therapy for hemophilia. J. Thromb. Haemost..

[36] Rosen S (2020). Activity of transgene-produced B-domain-deleted factor VIII in human plasma following AAV5 gene therapy. Blood.

[37] Zolotukhin I (2016). Potential for cellular stress response to hepatic factor VIII expression from AAV vector. Mol. Ther. Methods Clin. Dev..

[38] Poisson J (2017). Liver sinusoidal endothelial cells: Physiology and role in liver diseases. J. Hepatol..

[39] Olgasi C (2018). Patient-specific iPSC-derived endothelial cells provide long-term phenotypic correction of hemophilia A. Stem Cell Rep..

[40] Hollestelle MJ (2005). Extra-hepatic factor VIII expression in porcine fulminant hepatic failure. J. Thromb. Haemost..

[41] Webster WP (1971). Plasma factor VIII synthesis and control as revealed by canine organ transplantation. Am. J. Physiol..

[42] Madeira CL, Layman ME, de Vera RE, Fontes PA, Ragni MV (2009). Extrahepatic factor VIII production in transplant recipient of hemophilia donor liver. Blood.

[43] Lalor PF, Lai WK, Curbishley SM, Shetty S, Adams DH (2006). Human hepatic sinusoidal endothelial cells can be distinguished by expression of phenotypic markers related to their specialised functions in vivo. World J. Gastroenterol..

[44] Postic C, Magnuson MA (2000). DNA excision in liver by an albumin-Cre transgene occurs progressively with age. Genesis.

[45] Pham TH (2010). Lymphatic endothelial cell sphingosine kinase activity is required for lymphocyte egress and lymphatic patterning. J. Exp. Med..

[46] Kisanuki YY (2001). Tie2-Cre transgenic mice: A new model for endothelial cell-lineage analysis in vivo. Dev. Biol..

[47] Matsumura H, Hasuwa H, Inoue N, Ikawa M, Okabe M (2004). Lineage-specific cell disruption in living mice by Cre-mediated expression of diphtheria toxin A chain. Biochem. Biophys. Res. Commun..

[48] Ohmori T (2006). Efficient expression of a transgene in platelets using simian immunodeficiency virus-based vector harboring glycoprotein Ibalpha promoter: In vivo model for platelet-targeting gene therapy. FASEB J..

[49] Nishimura S (2015). IL-1alpha induces thrombopoiesis through megakaryocyte rupture in response to acute platelet needs. J. Cell Biol..

