# Supplementary material for: Characterization and visualization of murine coagulation factor VIII-producing cells in vivo
Source: Sci Rep. 2021 Jul 21;11:14824. doi: 10.1038/s41598-021-94307-0 (PMC8295325; doi:10.1038/s41598-021-94307-0)
Supplement: Supplementary file 1 — Supplementary Information 1. [file 41598_2021_94307_MOESM1_ESM.pdf]

Characterization and visualization of murine coagulation factor VIII-producing cells *in vivo*

Morisada Hayakawa,[1,2,*] Asuka Sakata,[3] Hiroko Hayakawa,[1] Hikari Matsumoto,[1] Takafumi Hiramoto,[1] Yuji Kashiwakura,[1] Nemekhbayar Baatartsogt,[1] Noriyoshi Fukushima,[4] Yoichi Sakata,[1] Katsue Suzuki-Inoue,[5] and Tsukasa Ohmori[1,2*]

[1]Department of Biochemistry, School of Medicine, Jichi Medical University, 3311-1 Yakushiji, Shimotsuke, Tochigi 329-0498, Japan

[2]Center for Gene Therapy Research, Jichi Medical University, 3311-1 Yakushiji, Shimotsuke, Tochigi 329-0498, Japan

[3]Center for Molecular Medicine, Jichi Medical University, 3311-1 Yakushiji, Shimotsuke, Tochigi 329-0498, Japan

[4]Department of Pathology, School of Medicine, Jichi Medical University, 3311-1 Yakushiji, Shimotsuke, Tochigi 329-0498, Japan

[5]Department of Clinical and Laboratory Medicine, Faculty of Medicine, University of Yamanashi, 1110 Shimokato, Chuo, Yamanashi 409-3898, Japan

***Corresponding Author:**

Morisada Hayakawa

Department of Biochemistry, School of Medicine, Jichi Medical University, 3111-1 Yakushiji, Shimotsuke, Tochigi 329-0498, Japan

Tel: +81-285-58-7324; Fax: +81-285-44-2158;

E-mail: morisada@jichi.ac.jp

***Co-Corresponding Author:**

Tsukasa Ohmori

Department of Biochemistry, School of Medicine, Jichi Medical University, 3111-1 Yakushiji, Shimotsuke, Tochigi 329-0498, Japan

Tel: +81-285-58-7324; Fax: +81-285-44-2158;

E-mail: tohmori@jichi.ac.jp

Supplemental Methods

Supplemental Figures: 5

Supplemental Table: 1

Description of Additional Supplemental Files

Supplemental Methods

Immunohistochemical analysis

Mice anesthetized with isoflurane were perfused with 50 mL PBS; then liver tissues were fixed with 10% formalin. Paraffin-embedded tissue samples were pretreated with 5% donkey serum and then treated with an anti-EGFP polyclonal antibody (code 598; MBL Co., Aichi, Japan) for 16 hours at 4°C. Immunoreactivity was detected with EnVision+ Dual Link System-HRP (Dako, Glostrup, Denmark) and 3,3′-diaminobenzidine (Agilent Technologies, CA, USA), followed by counterstaining with Myer hematoxylin. Tissue sections were observed with an all-in-one microscope (BZ-X710; KEYENCE, Osaka, Japan).

Isolation of hepatocytes and endothelial cells from liver of adult mice

C57BL/6J male mice were perfused with a perfusion buffer, and then livers were removed. After liver tissues were treated with collagenase/dispase and DNase I, the cells were separated by centrifugation at 50 × *g* for 2 minutes. From the precipitated cells, hepatocytes were isolated by 40% Percoll gradient centrifugation. From the supernatants, endothelial cells were separated by 25/50% Percoll gradient centrifugation. Next, $CD31^{high}CD146^{high}Lyve1^{+}$ endothelial cells were isolated using a BD FACSAriaII Special Order Research Product (BD Biosciences).

Quantitative reverse transcription-PCR

The total RNAs were isolated from hepatocytes and $CD31^{high}CD146^{high}Lyve1^{+}$ liver endothelial cells by TriPure Isolation Reagent (Sigma-Aldrich). Reverse transcription (RT) for first-strand cDNA synthesis was performed using a ReverTra Ace qPCR RT Master Mix with gDNA Remover (TOYOBO, Osaka, Japan). Real-time PCR was performed using

THUNDERBIRD SYBR qPCR Mix (TOYOBO) and Thermal Cycler Dice Real-Time System II (Takara Bio, Shiga, Japan). Reactions were analyzed in duplicate, and expression levels of the genes were normalized to *Hprt1* mRNA level. Primers used in the study are shown in the Supplemental Table 1.

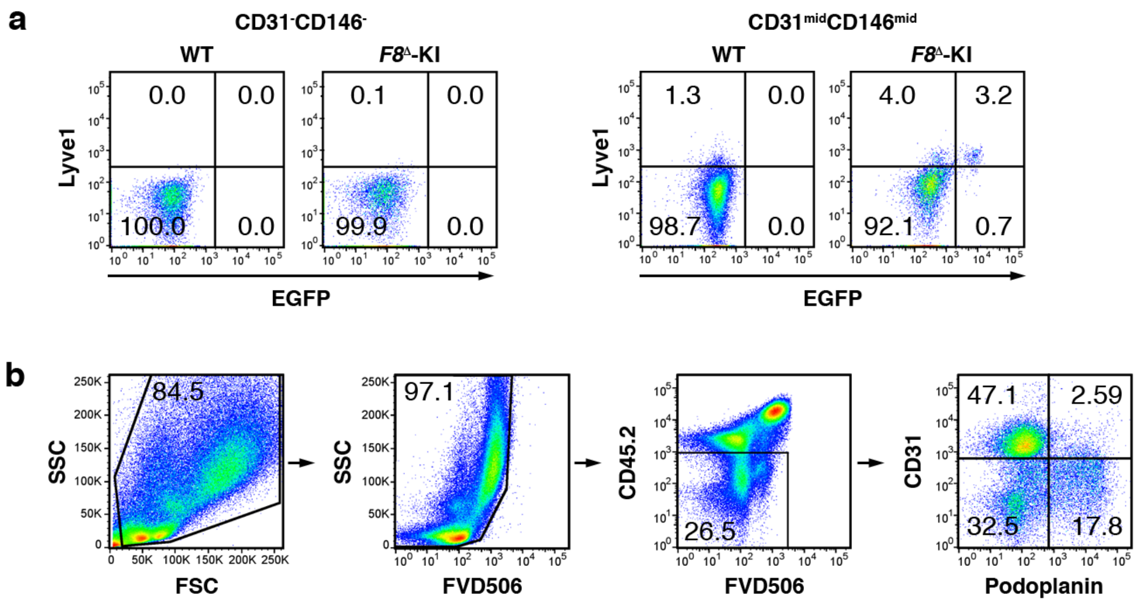

Figure S1: Expression of Lyve1 and Podoplanin in $F8^{\Delta}$ knock-in mice. a Plots represent the expression levels of EGFP (*horizontal*) and Lyve1 (*vertical*) in CD31^{-}CD146^{-} and CD31^{mid}CD146^{mid} liver cells obtained from WT and $F8^{\Delta}$-KI mice. **b** Lung cells were prepared from $F8^{\Delta}$ knock-in ($F8^{\Delta}$-KI) male mice. After dead cells and leukocytes were stained with FVD506 and CD45.2, expressions of podoplanin (*horizontal*) and CD31 (*vertical*) in the cells were determined by flow cytometry (n = 3).

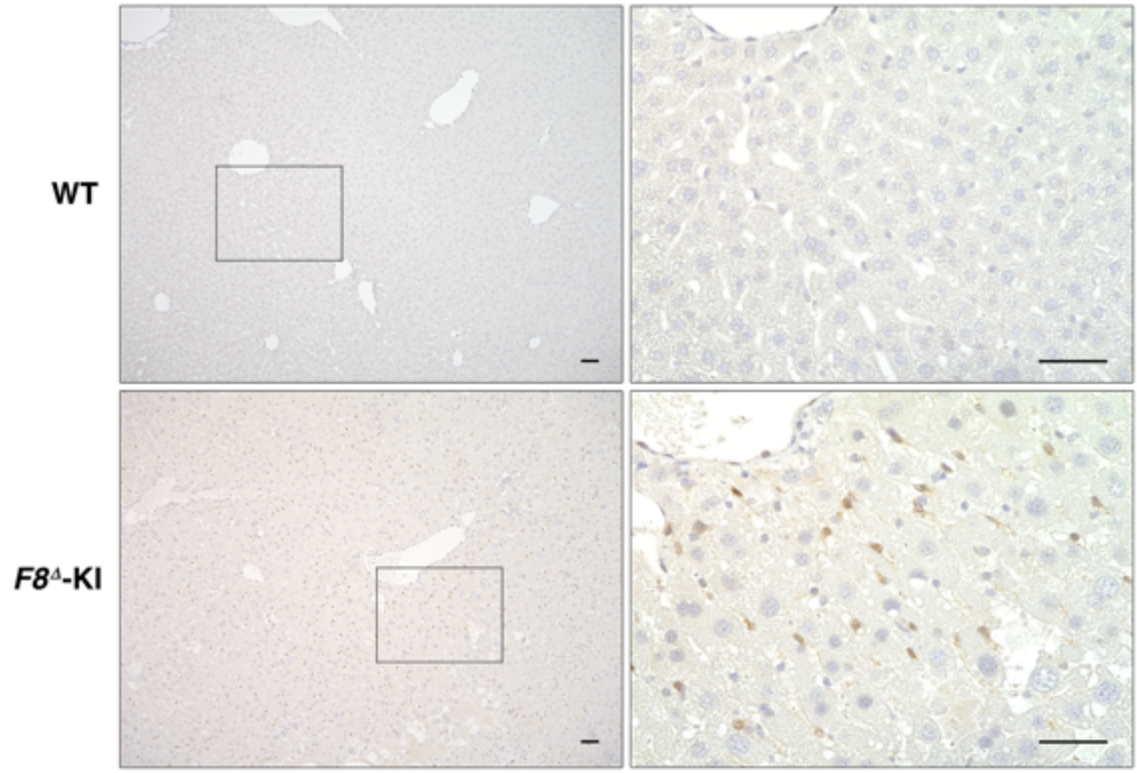

Figure S2: EGFP localization in *F8*$^{\Delta}$ knock-in mice. EGFP expression in liver sections obtained from wild-type C57BL/6J male mice (WT) and *F8*$^{\Delta}$-KI male mice was assessed by immunohistochemical analysis. The magnifications of the boxed areas in the left images are shown in the right panel. Brown-stained cells indicate EGFP-positive cells in *F8*$^{\Delta}$-KI mice. Scale bars: 50 μm.

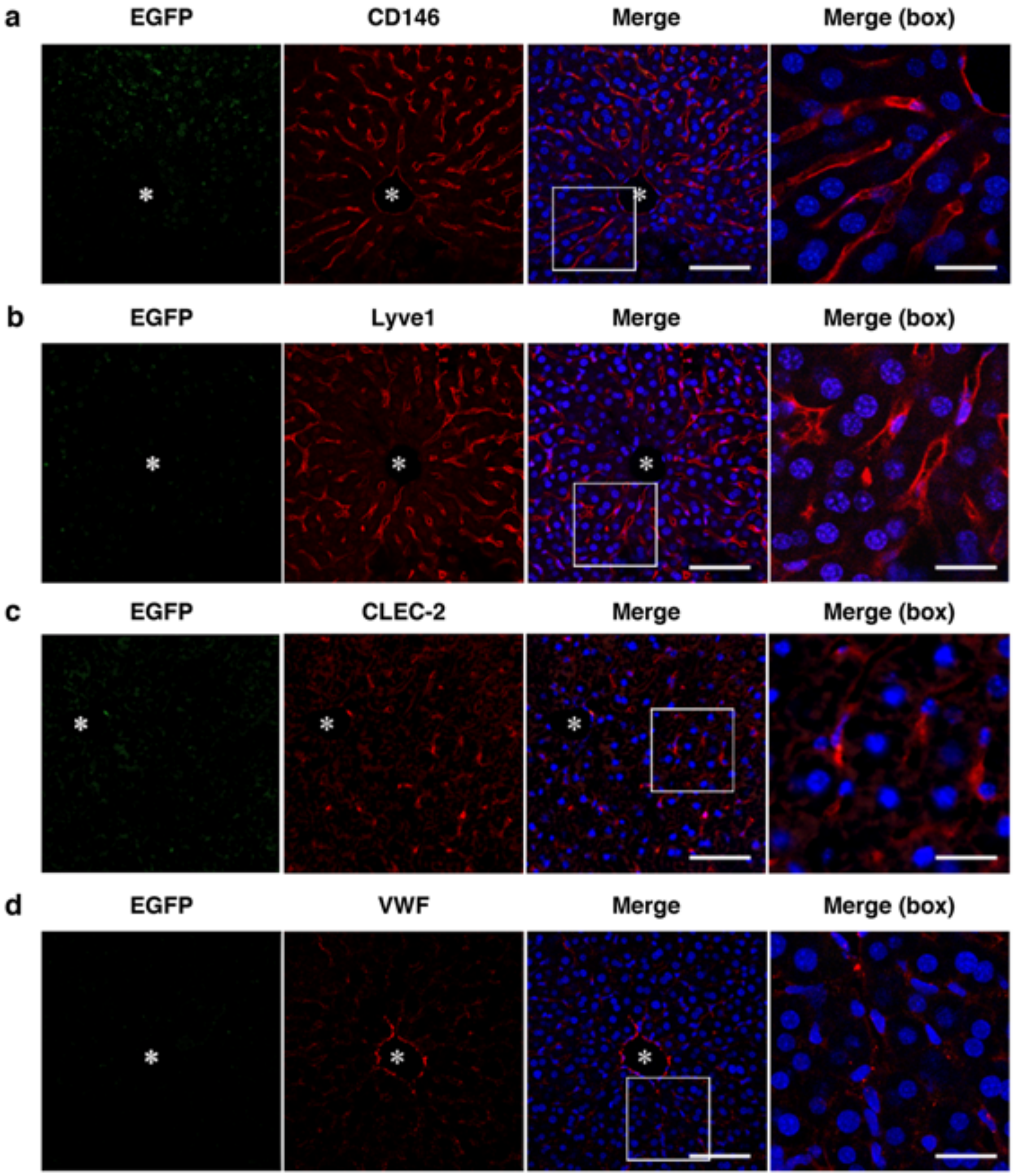

Figure S3: Localization of endothelial cell markers in C57BL/6J mice. (**a**–**d**) Liver sections obtained from adult C57BL/6J male mice were subjected to immunofluorescence staining for EGFP and CD146 **a**, EGFP and Lyve1 **b**, EGFP and CLEC-2 **c**, and EGFP and VWF **d**. Nuclear localization was simultaneously examined by DAPI staining. The magnification of the boxed areas in the merged image are shown in the right panel. Scale bars: 75 μm in merged images and 25 μm in magnification of the merged images; *, central vein.

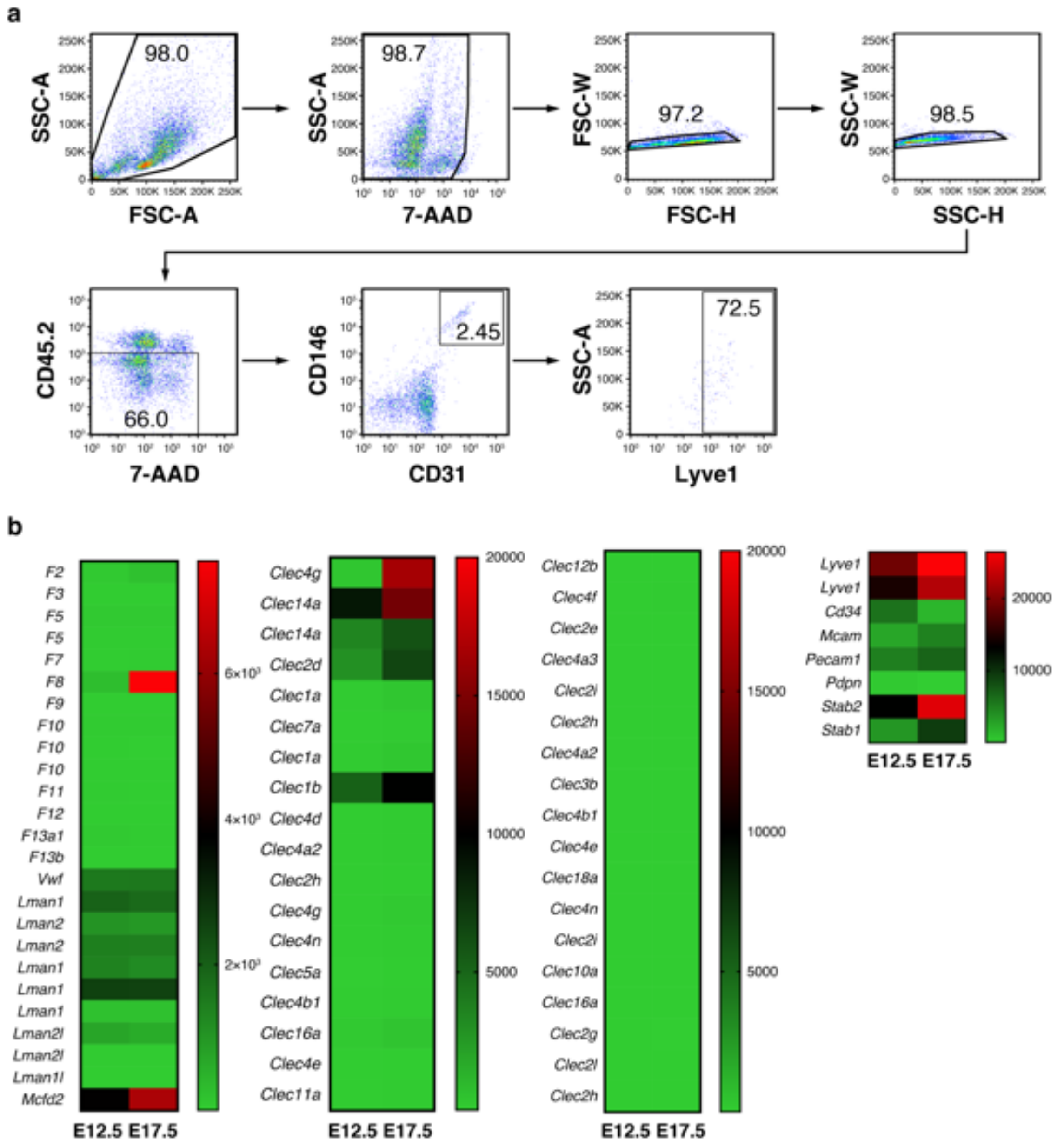

Figure S4: Gene expression profiles of Lyve1-positive cells in liver during embryonic development. **a** Gating strategy for sorting of CD31^{high}CD146^{high}Lyve1^{+} cells in C57BL/6J fetal liver. **b** Heatmap of differentially expressed genes related to coagulation factors and *Clec* family members between E12.5 Lyve1^{+} cells and E17.5 Lyve1^{+} cells (n = 3). Green and red colors represent low and high expression levels, respectively.

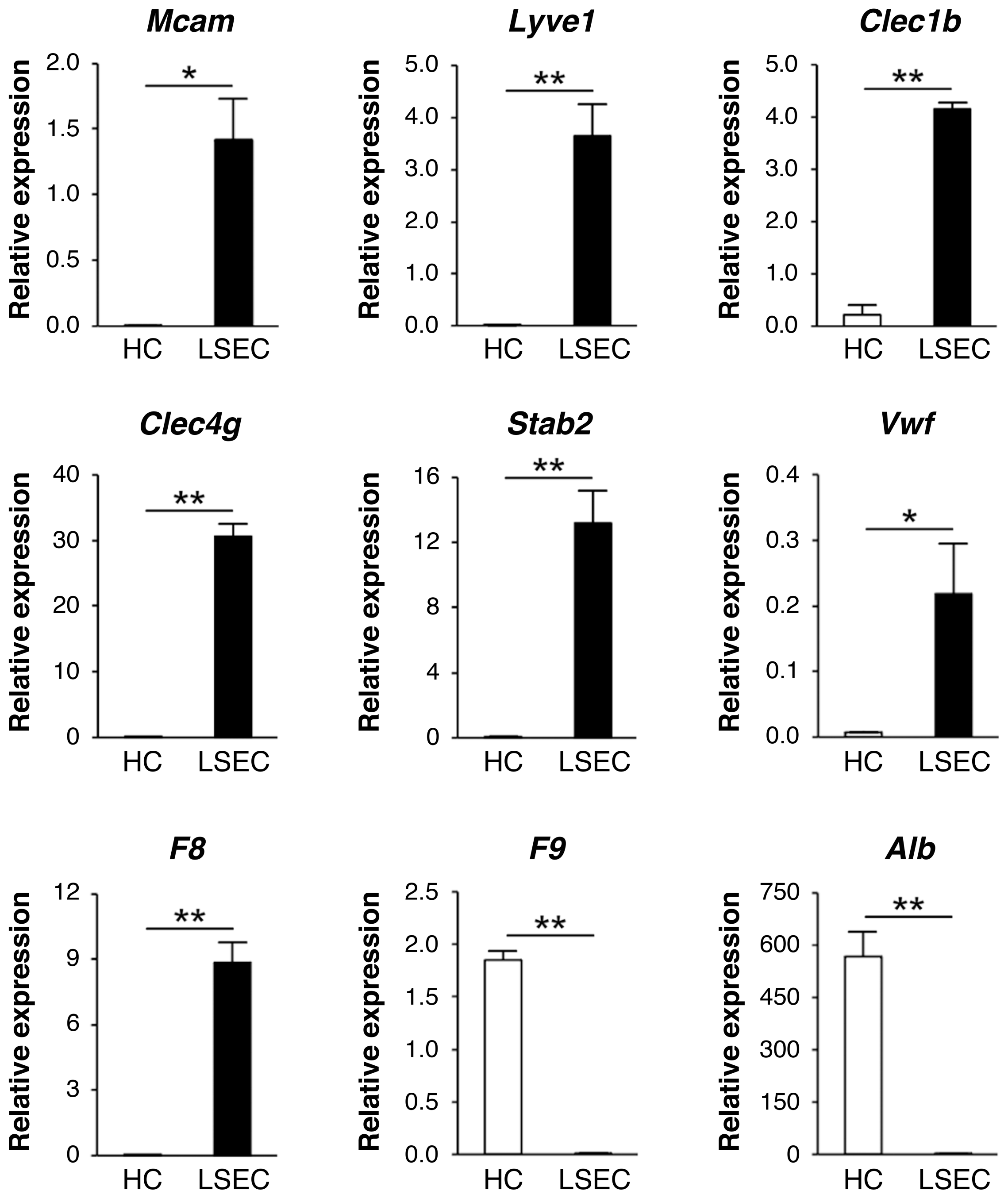

Figure S5: Expression of endothelial cell markers and coagulation factors in adult liver. Hepatocytes (HC) and $CD31^{high}CD146^{high}Lyve1^{+}$ liver sinusoidal endothelial cells (LSECs) were isolated from C57BL/6J adult mice. Expression of mRNA indicated genes was assessed by real-time RT-PCR and expressed as the fold increase versus each mRNA-to-*Hprt1* ratio. The data are shown as means ± SEM (n = 3). **P* < 0.05, ***P* < 0.01 (2-tailed Student's *t*-test).

Supplemental Table 1: Oligonucleotide primers for real-time qPCR.

Target genes	Sequence	
Mcam	F	5′-TTCCTGGCTTGAATCGTACC-3′
	R	5′-ACCCACACCTTCCTCTCCTT-3′
Lyve1	F	5′-CAGCATTCAAGAACGAAGCA-3′
	R	5′-GCAGCACCAAAGAAGAGGAG-3′
Clec1b	F	5′-GTTGTTGGACTCGTGGCTCT-3′
	R	5′-TTTCCTTCTCCGCCAGTAGA-3′
Clec4g	F	5′-CAAGCAAAGTTGATGGAGCA-3′
	R	5′-CGGATGTTCTCACGGTCTCT-3′
Stab2	F	5′-ATTGCCTCTAACGGGGTTCT-3′
	R	5′-CTGTTCCTAGGCCAGAGTGG-3′
Vwf	F	5′-GAATGCTGTGGGAGATGCTT-3′
	R	5′-GCCATCCTGGAATGTCTCAT-3′
F8	F	5′-GTTTGCACCCCACTCATTCT-3′
	R	5′-TCCATTCCCAATGGTATGCT-3′
F9	F	5′-ATGAATGCTGGTGCCAAGTT-3′
	R	5′-TGCACCTGCCATTTTTAATGT-3′
Alb	F	5′-GCTGAGACCTTCACCTTCCA-3′
	R	5′-CTTGTGCTTCACCAGCTCAG-3′
Hprt1	F	5′-AGCAGTACAGCCCCAAAATG-3′
	R	5′-CGAGAGGTCCTTTTCACCAG-3′

Description of Additional Supplemental Files

File Name: Supplemental Movies 1

Description: Intravital imaging in the liver of WT mouse. *Red*, blood flow; *blue*, Hoechst-stained nuclei

File Name: Supplemental Movies 2

Description: Intravital imaging in the liver of $F8^{\Delta}$-KI mouse. *Red*, blood flow; *blue*, Hoechst-stained nuclei; *green*, EGFP

Uncropped images of Southern blots in Figure 1b and agarose gel in Figure 1c

Figure 1b

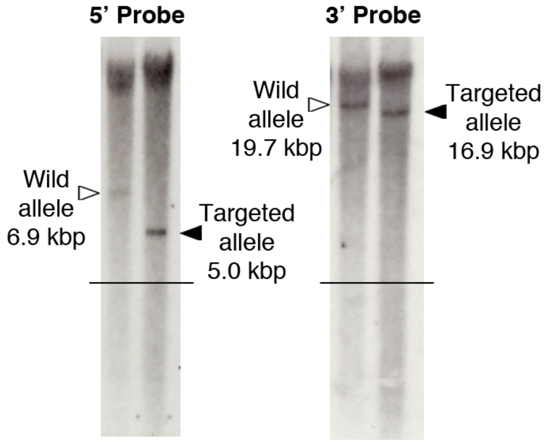

Figure 1c

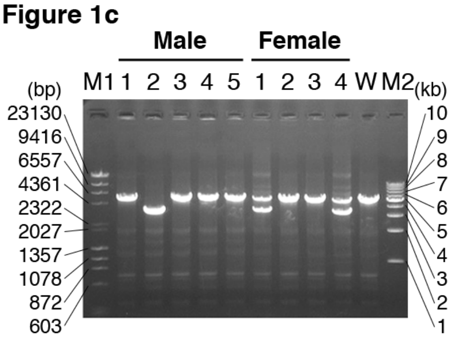

M1, λHindIII digest + ϕX174HaeIII digest mix marker
M2, 1 kb ladder maker

Wild allele, 5037 bp
Deleted allele, 3494 bp

Since the generation of FVIII knock-in mice was outsourced, the Southern blot images in Figure 1B were based on data from the company's report below.

*** The Japanese part of the report is translated into English in red letters.**

Southern blot Genotyping 結果 (5', 3' probe)

Results of Southern blot Genotyping (5', 3' probe)

(試験目的) (Subject)

Targeting Vectorによる相同組換えが目的の配列で正しく起こっていることを確認した。

We confirmed that the homologous recombination by Targeting Vector occurred correctly in the target sequence.

(解析方法) (Methods)

Short armもしくはlong armの外側で切断する制限酵素を用いてゲノムを消化した。
そして、5'側及び3'側で作製したprobeを用いて、Wild Type allele及びTargeted alleleの2本のバンドを検出することで、5', 3'側相同組換えを確認した。

The genome was digested with restriction enzymes that cut outside the short arm or long arm.
The 5' and 3' side homologous recombination was then confirmed by detecting two bands of Wild Type allele and Targeted allele using the probes on the 5' and 3' sides.

(ES Sample)

PCR Genotypingにより陽性と判定された1clone

One clone identified as positive by PCR genotyping

clone No. 11

(制限酵素処理)

5' ・・・ ApaL I (WT allele/Targeted allele = 6.9kbp/5.0kbp)

3' ・・・ Sph I (WT allele/Targeted allele = 19.7kbp/16.9kbp)

(Probe)

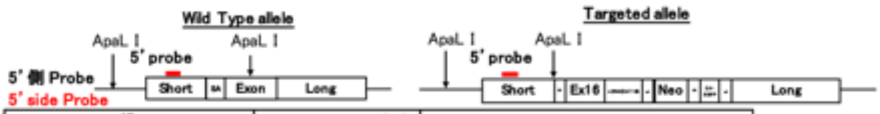

ID	Product length (bp)	Sequence
5'-No.1 probe primer (Forward)	336	AAGGTATGAGGCAAGGCATTGATA
5'-No.1 probe primer (Reverse)		CACATACTCATGATAAAGTAATGAACA

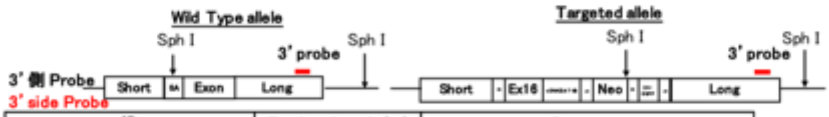

ID	Product length (bp)	Sequence
3'-No.1 probe primer (Forward)	406	AAGATAATTTCAAGGGTGCTATTCAG
3'-No.1 probe primer (Reverse)		GGTAAAGGAATGAGATAAAGTGCAA

(結果) (Results)

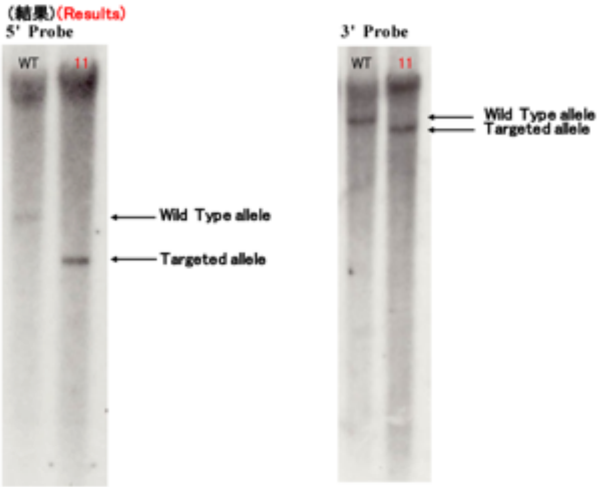

clone No. : 陽性バンドが検出されたclone No.
: Clone No. indicates that a positive band was detected.
WT : Wild Type genome

5', 3'側相同組換え陽性clone No. : 11 (計1clone)

Positive clone No. in 5', 3' side of the homologous recombination : 11 (counting 1 clone)
